# Effect of physical fitness on colorectal tumor development in patients with familial adenomatous polyposis

**DOI:** 10.1097/MD.0000000000017076

**Published:** 2019-09-20

**Authors:** Tomiyo Nakamura, Hideki Ishikawa, Toshiyuki Sakai, Makoto Ayabe, Keiji Wakabayashi, Michihiro Mutoh, Nariaki Matsuura

**Affiliations:** a Department of Molecular Pathology, Osaka University Graduate School of Medicine & Health Science, Osaka; b Department of Food Sciences and Human Nutrition, Ryukoku University, Shiga; c Department of Molecular-Targeting Cancer Prevention; d Department of Drug Discovery Medicine, Kyoto Prefectural University of Medicine, Kyoto; e Department of System Human Information Engineering, Okayama Prefectural University, Okayama; f Graduate Division of Nutritional and Environmental Sciences, University of Shizuoka, Shizuoka; g Division of Prevention, Center for Public Health Sciences, National Cancer Center,Tokyo, Japan.

**Keywords:** colorectal adenomas, colorectal cancer, cross-sectional study, familial adenomatous polyposis, maximal oxygen uptake, physical fitness

## Abstract

Although accumulated epidemiological evidence indicates that a good physical fitness level may prevent the development of sporadic colorectal cancer (CRC), few studies have examined the effect of physical fitness level on familial adenomatous polyposis (FAP). This cross-sectional study aimed to examine the relationship between physical fitness and CRC development in patients with FAP.

A total of 119 patients (54 male; 65 female) with FAP, aged 17 to 73 years, underwent a step test to induce exercise stress. Predicted maximal oxygen uptake (VO_2_max) was calculated for each patient by using heart rate as an index of physical fitness. The association of VO_2_max with the presence or absence of CRC and polyp diameter was examined. Patients with FAP were divided into 3 categories according to their VO_2_max (high, medium, and low). The association between maximum polyp size and VO_2_max among the patients with FAP without a history of colectomy was examined.

The risk of CRC was significantly higher in the low VO_2_max group than in the high VO_2_max group (odds ratio = 4.07; 95% confidence interval, 1.02–16.26). The maximum polyp diameter was significantly negatively correlated with the VO_2_max among the patients with FAP without a history of colectomy (*r* = −.44, *P* = .01). In the multiple linear regression analysis, maximum polyp diameter was independently correlated with VO_2_max.

Our results suggest a preventive association between physical fitness and CRC development or colorectal adenoma growth exists in patients with FAP.

## Abstract 2

Supplemental Digital Content is available in the text

## 1 Introduction

In Japan, the prevalence of colorectal cancer (CRC) is not as high as that in the United States and Europe; however, the incidence rates are increasing with the westernization of Japanese people's lifestyles.^[1,2]^ Several proactive studies in Japan have focused on the prevention of CRC through lifestyle interventions.^[3,4]^ Epidemiological studies on the lifestyle-related factors associated with the occurrence of sporadic CRC have provided compelling evidence on the effect of exercise and physical fitness on the prevention of this type of cancer.^[5–7]^ However, few studies have used exercise stress tests to measure physical fitness.^[8,9]^

Familial adenomatous polyposis (FAP), characterized by an ultra-high risk of CRC,^[10]^ is a rare genetic disease usually caused by a pathological mutation of the *APC* gene in the germ line. Patients with FAP develop multiple colorectal adenomas at a young age and begin to develop CRC at around the age of 20 years; eventually, about half of all these patients develop cancer by the age of 40 years.

The prevalence of FAP is considered to range from 1 in 10,000 to 1 in 20,000 people worldwide, and is approximately 1 in 17,400 persons in Japan.^[10]^ As Japan's population was approximately 126,420,000 people in 2018,^[11]^ the proportion of patients with FAP was estimated to be around 7300.^[2,10]^

The main etiology of FAP is a genetic mutation in the germline, suggesting limited involvement of environmental factors in cancer development. At the same time, the density of colorectal adenomas and the timing of malignant transformation of adenomas vary across people in the same family, suggesting a certain involvement of environmental factors.^[12]^ Moreover, the suggested similarity between the pathogeneses of CRCs in FAP patients and sporadic CRC^[6,13]^ indicates the influence of physical activity on cancer development in FAP. A recent systematic review and Bayesian dose–response meta-analysis^[14]^ suggested that total physical activity levels several times higher than the current recommended minimum level can significantly reduce the risk of colon cancer. Animal experiments^[15,16]^ have shown that participation in moderate-intensity exercise training, such as treadmill training, alters cellular pathways and decreases the number and size of intestinal polyps in Apc (Min/+) mouse. Therefore, higher physical activity levels that increase maximum oxygen uptake may reduce polyp number and size in patients with FAP. However, to the best of our knowledge, no study performed to date has examined the relationship between maximal oxygen uptake (VO2max) and the development of CRC or adenoma in these patients. Hence, we aimed to examine the relationship between physical fitness and CRC development in patients with FAP

## 2 Materials and methods

### 2.1 Study population

In this cross-sectional study, we enrolled patients who had participated in 2 previous CRC prevention studies (J-FAPP study and J-FAPP study II) and had undergone a step test. Both the aforementioned studies were double-blind, randomized clinical trials that were conducted as part of the Third Cancer Conquest Strategic Program of the Ministry of Health, Labour and Welfare of Japan. Both studies had a multicenter design and targeted patients with FAP who were treated on an outpatient basis at medical institutions across the country. All participants enrolled in these trials received nutritional and exercise plans, including a step test and physical fitness assessment. The details of these trials have been described elsewhere.^[17,18]^

The participants in the present study included Japanese patients with FAP aged 16 years or older.

The inclusion criteria were as follows^[17,18]^:

(1) Patients with FAP
(2) Patients with ≥100 colorectal adenomas
(3) Patients with or without a pathological mutation in the APC gene in the germline, as detected through protein truncation testing^[2]^

The exclusion criteria were as follows^[17,18]^:

(1) Patients with active cancer at the time of recruitment
(2) Patients currently taking an antithrombotic or anticoagulant agent
(3) Patients with a history of stroke or gastric or duodenal ulcers (except for those with healed scars, as confirmed after the successful eradication of *Helicobacter pylori*)
(4) Patients with inflammatory enteritis, hemorrhagic diverticulitis, or bleeding tendency
(5) Patients with a platelet count of ≤100,000 cells/mm^3^ and with an abnormal prothrombin time
(6) Patients with a known allergy to aspirin
(7) Patients who were pregnant or planned to become pregnant during the trial period
(8) Patients taking nonsteroidal anti-inflammatory drugs for pain relief >3 times a week

The participants recruited in the present study were approached across Japan. They participated in interviews and were referred to us either by a physician-in-charge who was aware of the present study or they contacted us directly. Participants treated by HI (the principal investigator) as the physician-in-charge were also included. If a patient requested an interview in an affiliated medical institution with an active ethics committee, an assigned physician-in-charge interviewed the patient. In other institutions, HI would visit the patient for an in-person interview upon receiving a request from the physician-in-charge, after approval was obtained from the appropriate ethics committee. Informed consent was obtained, and after schedule adjustments with the patient, a step test was conducted to collect preintervention data. At the same time, we documented data on patients’ height, weight, medical history, medication history, drinking habits, current smoking status, lifestyle, physical activity, and the use of nonsteroidal anti-inflammatory drugs, such as aspirin. Most patients participating in this study were treated at unaffiliated medical institutions (36 in total).

The study was approved by the ethics review committee of Osaka Medical Center for Cancer and Cardiovascular Diseases (ethics application submitted 1999.11) as well as by the ethics committees of all the participating medical institutions.

### 2.2 Colonoscopy

Data on the occurrence of CRC, surgery, and colonoscopic findings were collected from the participants’ medical records. Data on cancer development were collected up to the time physical fitness was measured in this study. Polyp diameter was measured using biopsy forceps or measuring forceps during colonoscopy. During colonoscopic examinations, endoscopists checked for the presence of cancer in any of the numerous polyps as the top priority. If no cancer was found, large polyps were examined.^[19]^ Endoscopy was usually performed by 2 endoscopists. Changes in the maximum polyp size and number of polyps were assessed blindly by the Data Evaluation Committee by comparing patients’ endoscopic photographs.^[17,18]^

Our study included patients with FAP who had undergone multiple colonoscopic examinations, but not colectomy. Among the polyps found in these colonoscopic examinations, the diameter of the largest polyp (excluding that of carcinomas) was taken as the maximum polyp diameter. Patient age was the one recorded at the step test. The endoscopists were blinded to the patients’ step test results, and the physician who performed the step test was blinded to the patients’ cancer status.

### 2.3 Predicted maximal oxygen uptake

The participants underwent an exercise stress test (a step test) twice between September 2000 and August 2007, one at the time of recruitment and another 2 years later. Of the 47 prefectures in Japan, 16 prefectures including the northernmost part (Hokkaido) and the southernmost part (Kyushu) took part in this study. The step test used in this study was the exercise stress test developed at Fukuoka University in Japan.^[20,21]^ In the step tests, all measurements were performed by 1 physician (HI) and 1 trained assistant. Measurements were performed in all participants employing the same procedures.

The step test was conducted in a private room, either in a participating medical institution or a health center. The exercise stress test was performed at least 2 hours after the last meal and after confirming resting blood lactic acid (LA) levels to be ≤1.5 mmol/L. The step test was performed using a 20-cm-high step with multistage intensities, comprising three 4-minute exercises and 2-minute rest cycles. Heart rate (HR) was measured immediately after the 4-minute exercise test. Each step exercise was performed in step with the beats of a metronome. Exercise stress intensity was calculated from the number of steps and the metronome beat rates with 3.9 metabolic equivalents (METs) for 15 steps/min, 4.9 METs for 20 steps/min, 5.8 METs for 25 steps/min, 6.8 METs for 30 steps/min, and 7.8 METs for 35 steps/min. Participants younger than 50 years underwent the exercise stress tests at 3 intensity levels, namely, 20, 25, and 30 steps/min and those aged ≥50 years underwent the test at 15, 20, and 25 steps/min. If the LA level had not increased at the third intensity level, the participants underwent an exercise stress test with a fourth intensity level (35 or 30 steps/min). VO2max was calculated based on the increase in HR at the point where the exercise stress intensity in the step test increased from 5.8 to 6.8 METs by using the following formula^[22]^: 
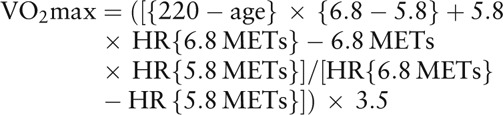


### 2.4 Statistical analysis

Continuous variables were expressed as median and interquartile range and were compared by the presence or absence of CRC by using a Mann–Whitney *U* test. Categorical variables were presented as frequency (percentage), and were analyzed using a *χ*^2^ test.

Regarding the relationship between physical fitness and CRC,^[23,24]^ data were adjusted for factors potentially related to the participants’ fitness level, such as age (continuous), sex, body mass index (BMI) (continuous), *APC* gene mutation, age at FAP diagnosis (continuous), history of other cancers or diseases (hypertension, diabetes, dyslipidemia, and so on), history of colectomy, alcohol intake (never, <3 times/week, and ≥3 times/week), and smoking status (non-smoker, current smoker, and former smoker), using logistic analysis. Patients with FAP usually undergo colectomy about 2 months after diagnosis. Thus, the age at FAP diagnosis was nearly equal to that at which many of the participants underwent colectomy. The patients with FAP were divided into tertiles according to their VO2max levels (VO2max) (high, medium, and low). Participants with “high” VO2max were assigned to the reference category.

As it is widely known, the risk of cancer onset is higher in cases with a colon polyp diameter exceeding 10 mm.^[25]^ Hence, the relationship between maximum polyp size and VO_2_ max among the patients with FAP without a history of colectomy was examined. Correlations were used to assess univariate relationships between the variables, whereas regression was used to assess independent associations. All statistical tests were 2-sided, and a significance level of 5% was used in all analyses. Results were analyzed using SPSS for Windows software package version 23.0 (IBM Corp, Armonk, NY).

## 3 Results

**Figure 1:**
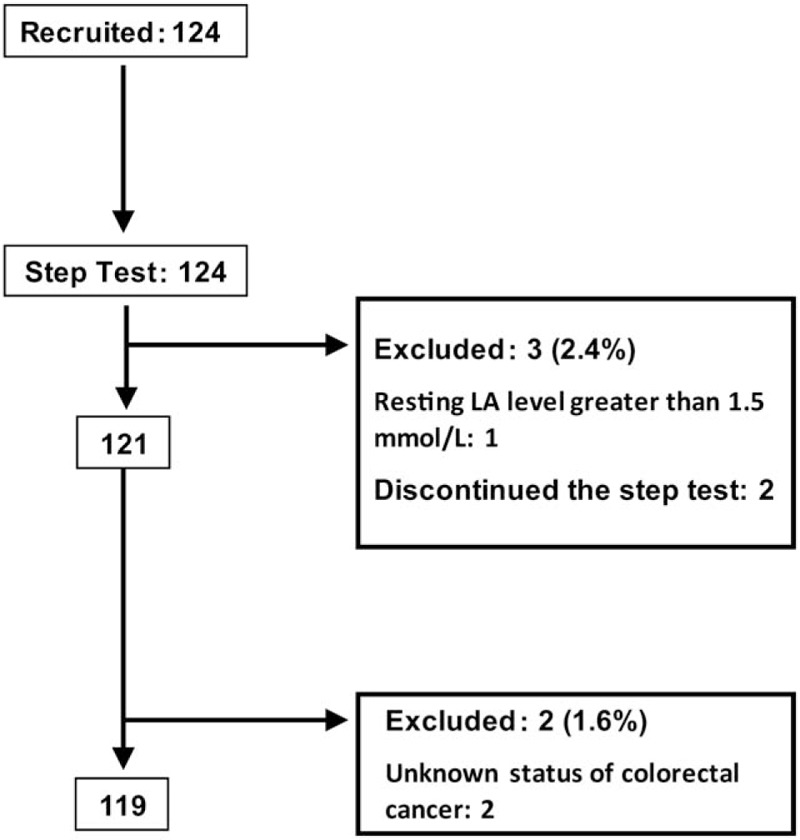
Flow chart of the study procedure. LA = lactic acid.

We approached 141 potential participants, of which 124 (87.9%) agreed to participate in the study, and underwent the step test. After the exclusion of 5 patients, including 1 patient who had a resting LA level >1.5 mmol/L before the stress test, 2 patients who discontinued the test because of physical deconditioning during the first and second stages of the test, and 2 patients with unknown status of CRC after surgery, the remaining 119 patients (54 male; 65 female) were included in the analysis (Fig. 1).

The participants’ characteristics according to sex and history of CRC are summarized in Table 1. The age range of the participants was 17 to 73 years. No significant difference in sex was observed. The age, age at FAP diagnosis (females only), history of other diseases, and history of colectomy were significantly higher in patients with a history of CRC than in those without. No significant difference was observed between these patients in terms of BMI, FAP diagnosis, *APC* gene mutation, history of other cancers, alcohol intake, or smoking status.

**Table 1:**
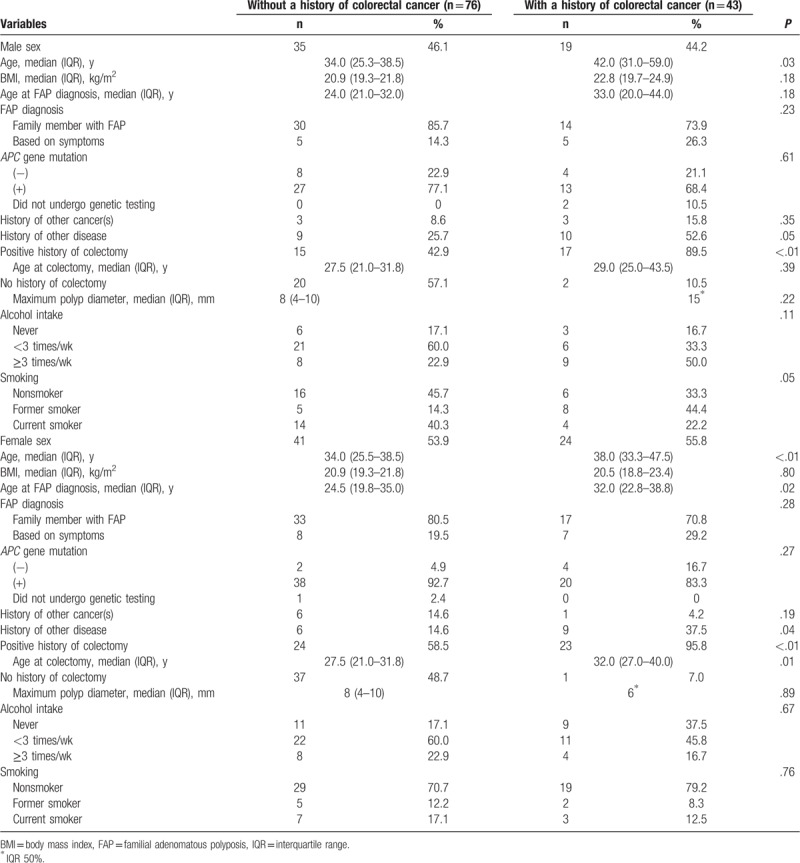
Characteristics of the participants in our study according to their history of colorectal cancer.

The diagnosis of FAP was prompted by various factors. Some patients (n = 94) underwent genetic or colonoscopic examinations as one of their parents had FAP, whereas other patients (n = 25) were diagnosed based on symptoms such as melena, abdominal pain, and anemia. A total of 116 (97.5%) patients underwent genetic testing, and 98 (84.5%) had a pathological mutation in the *APC* gene in the germline. Across these groups, there were no significant differences related to the history of CRC.

The difference in age is the most important potential confounder. The VO_2_max declined with age (Supplemental Figure 1); however, analysis by age stratification also showed that VO2max tended to be low in patients with a history of CRC. In patients without a surgical history, the VO_2_max was significantly higher in those without a history of CRC (data not shown).

In 10 cases, 2 members of the same family were included, and in 7 cases, 3 members of the same family were included (Supplemental Figure 2). We also performed analyses according to family line, but no differences were observed in the results. Therefore, family line-related adjustments were not made in the analysis. The mean period from the first to the next CRC diagnosis was 3.8 years (range: 1–16 years), and the mean period from the initial CRC diagnosis to the step test was 6.3 years (range: 0–21 years).

Table 2 shows the odds ratio (OR) of the association between history of CRC and VO2max. OR1 was adjusted only for age and sex, and OR2 adjusted for factors potentially related to fitness level. The risk was significantly higher in the low VO2max group than in the high VO_2_max group (OR2 = 3.32; 95% confidence interval, 1.00–11.02).

**Table 2:**
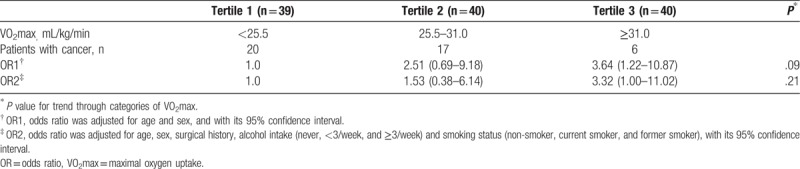
Odds ratios and their 95% confidence intervals for history of colorectal cancer according to the maximal oxygen uptake value.

**Figure 2:**
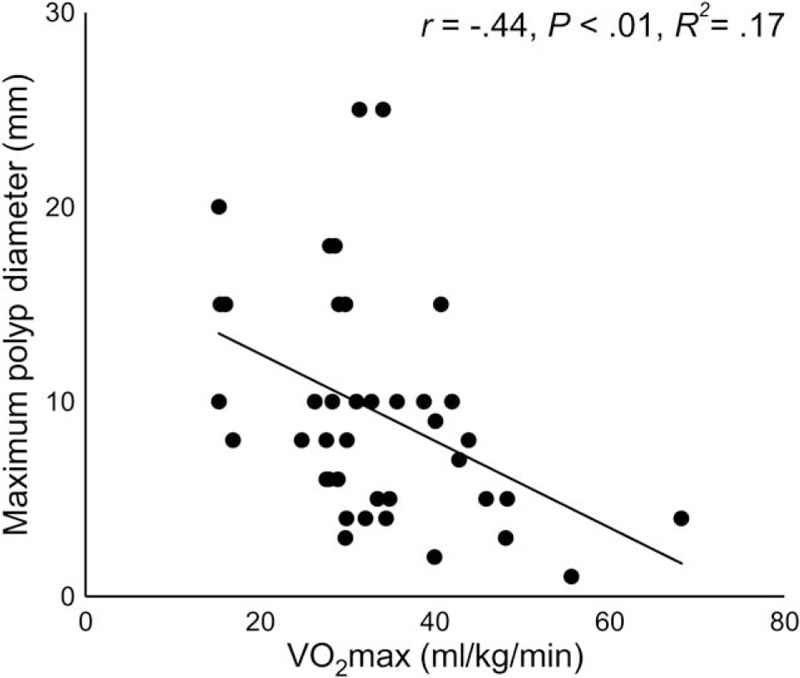
Relationship between age and maximal oxygen uptake. VO_2_max =  maximal oxygen uptake.

The date on which the polyp with the maximum size among all found those was observed ranged from 3 years before the step test to 2 years after the step test. In this study, polyps with the maximum size were found during the first colonoscopic examination performed after FAP diagnosis in more than half (57.5%) of the participants. Figure 2 shows the correlation between maximum polyp diameter and VO2max among 40 patients without a previous history of colectomy (22 maleds and 18 females). Maximum polyp diameter showed a significant negative correlation with VO_2_max (*r* = −0.44, *P* < .01), and was independently correlated with VO_2_max in the multiple linear regression analysis (Table 3).

**Table 3:**
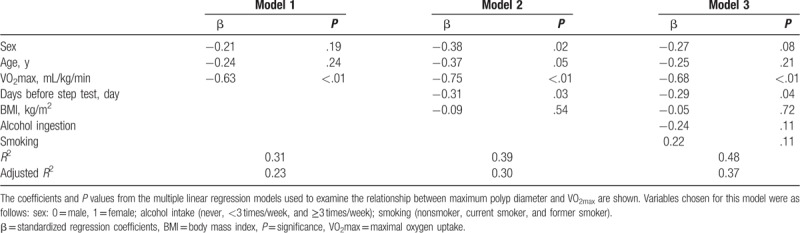
Relationship between maximum polyp diameter and maximal oxygen uptake among the patients with FAP without a surgical history of colectomy (n = 40).

## 4 Discussion

A number of epidemiological studies have provided compelling evidence that exercise (physical activity) can reduce the risk of sporadic CRC.^[3–5]^ In the present study we observed that in patients with FAP, the risk of CRC was significantly higher in the low VO_2_max group than in the high VO_2_max group. There was a significant negative correlation between maximum polyp diameter and VO_2_max among the patients with FAP without a history of colectomy. These observations suggest that physical fitness plays a role not only in the development of sporadic CRC, but also in cancer development in FAP. The results of the present study also indicate that physical fitness affects the development of colorectal adenoma, a precancerous lesion.

Some studies on sporadic CRC have suggested that participation in active physical activity of a moderate-to-high intensity for 30 to 60 min/day may reduce the risk of CRC, and that the risk of CRC among patients with high levels of physical activity declines with an increase in the amount of physical activity.^[24]^ A meta-analysis also showed that physical activity reduces the risk of colorectal polyps and strongly suppresses polyp growth.^[25]^ As a higher level of physical activity is likely to lead to greater physical strength, it is likely that physical fitness has a similar association with sporadic CRC and cancer development in FAP. The mechanism by which physical fitness prevents the development of CRC and adenoma is largely unknown, although the involvement of improved insulin resistance and altered intestinal transit time has been suggested.^[26]^ Many of those studies evaluated the effect of exercise and physical fitness using a questionnaire survey.^[24,25]^ As we evaluated physical fitness by VO_2_max, we believe the results of our study to be more accurate.

We assumed the selection bias between the participants and the general Japanese patients with FAP to be small based on the following considerations: the participants were not recruited under specific conditions, the number of patients who refused to participate was small, and a relatively large number of patients were enrolled despite the rarity of FAP occurrence. As the step tests were performed at different locations and in different seasons, the possible effect of these factors was considered. To minimize these effects, we performed the step tests in a private room in which patients could be at ease, and after confirming a low blood LA level. The endoscopists who measured polyp diameters were blinded to the patients’ step test results, and the physician who performed the step test was blinded to the patients’ cancer status. However, as noted above, the step test yields are extremely objective values that are considered to be minimally influenced by the knowledge of a patient's cancer status.

Among the participants who refused surgery, large polyps found on colonoscopic examinations were resected endoscopically. Therefore, the results of this study may have been influenced by endoscopic resection performance. However, polyps with the maximum size were observed in the first colonoscopic examination performed after FAP diagnosis in more than half of the patients, and the results in such patients were thus not considered to have been influenced by endoscopic resection.

Our study has several limitations. First, it had a cross-sectional design; therefore, causality could not be established between fitness and colorectal polyp development. Second, the age was significantly higher in the patients with CRC than in those without CRC. Therefore, the risk was adjusted for age, sex, and other related factors. However, the tendency did not change even after adjustment. Another limitation was the estimation of the VO2max by using the HR instead of the LA level. The use of HR is associated with an increased error in the estimation of the VO2max as it is influenced by factors such as heart diseases.^[27]^ However, considering the similarities in the prevalence rates of heart diseases between patients with FAP and the general population, and the fact that many patients in this study were young, we believe that the effect of this factor is limited. Another limitation is the inclusion of patients who had developed cancer and those who had undergone surgery. However, it is unlikely that the presence of polyps was associated with reduced physical fitness as similar results were obtained even when only the patients who had undergone surgery were included in the analysis. Moreover, the evaluation of the maximum polyp diameter in patients without a history of surgery indicated that the presence of polyps did not necessarily induce symptoms or affect the activities of daily living.

Finally, it is not clear how polyp size can be measured precisely using endoscopy. However, as mentioned above, information on maximum polyp diameter is clinically important. Therefore, it is necessary for polyp diameter to be measured precisely using forceps or snares. In addition, as the participants’ fitness levels were not ascertained at the time of polyp size measurement, the fitness test results were unlikely to have influenced polyp diameter measurement.

We believe that our study makes a significant contribution to the literature because no study performed to date has examined the relationship between Vo2max and CRC or adenoma development in patients with FAP. However, it is necessary to conduct a prospective cohort study or an intervention study to verify whether CRC development or tumor growth can be prevented through instructed exercise in patients with FAP.

## Acknowledgments

The authors express their gratitude to all the primary physicians of the patients with FAP and to Ms. Tomoko Saeki, an administrative assistant, for her assistance in the step test performance and data entry, and Mrs. Ikuko Takeyama, a dietitian, for her assistance in collection of dietary data and exercise data. The authors thank Dr. Hiroaki Tanaka who planned and supported this research but who unfortunately passed away last year.

## Author contributions

**Conceptualization:** Hideki Ishikawa.

**Data curation:** Hideki Ishikawa.

**Formal analysis:** Tomiyo Nakamura.

**Funding acquisition:** Hideki Ishikawa.

**Investigation:** Tomiyo Nakamura, Hideki Ishikawa.

**Methodology:** Hideki Ishikawa.

**Project administration:** Hideki Ishikawa.

**Software:** Makoto Ayabe.

**Supervision:** Toshiyuki Sakai.

**Validation:** Makoto Ayabe.

**Visualization:** Tomiyo Nakamura.

**Writing – original draft:** Tomiyo Nakamura.

**Writing – review & editing:** Keiji Wakabayashi, Michihiro Mutoh, Nariaki Matsuura.

Tomiyo Nakamura orcid: 0000-0003-4069-9779.

## Supplementary Material

Supplemental Digital Content
