# Supplementary material for: Effect of physical fitness on colorectal tumor development in patients with familial adenomatous polyposis
Source: Medicine (Baltimore). 2019 Sep 20;98(38):e17076. doi: 10.1097/MD.0000000000017076 (PMC6756628; doi:10.1097/MD.0000000000017076)
Supplement: Supplemental Digital Content [file medi-98-e17076-s001.docx]

**Supplementary Material**

**

**

**Supplemental Figure 1.** Relationship between maximum polyp diameter and maximal oxygen uptake.

VO_2_max = maximal oxygen uptake

**

**

**Supplemental Figure 2.** Relationship between age and maximal oxygen uptake according to the number of members in individual families.

VO_2_max = maximal oxygen uptake
